# Intrinsic and Tumor Microenvironment-Induced Metabolism Adaptations of T Cells and Impact on Their Differentiation and Function

**DOI:** 10.3389/fimmu.2016.00114

**Published:** 2016-03-29

**Authors:** Soumaya Kouidhi, Muhammad Zaeem Noman, Claudine Kieda, Amel Benammar Elgaaied, Salem Chouaib

**Affiliations:** ^1^Laboratory BVBGR, LR11ES31, ISBST, Higher Institute of Biotechnology of Sidi Thabet, University of Manouba, Tunis, Tunisia; ^2^Laboratory of Genetics, Immunology and Human Pathology, Faculty of Sciences of Tunis, University Tunis El Manar, Tunis, Tunisia; ^3^Laboratory «Integrative Tumor Immunology and Genetic Oncology» Equipe Labellisée LIGUE 2015, Institut National de la Santé et de la Recherche Médicale (INSERM) UMR1186, Villejuif, France; ^4^Institut National de la Santé et de la Recherche Médicale (INSERM), Gustave Roussy, Univ. Paris-Sud, Université Paris-Saclay, Villejuif, France; ^5^Centre de Biophysique Moléculaire, CNRS UPR 4301, Orléans, France

**Keywords:** immune system, T lymphocytes, tumor cell metabolism, cancer, hypoxia, tumor microenvironment

## Abstract

It is well recognized that the immune system and metabolism are highly integrated. In this context, multilevel interactions between metabolic system and T lymphocyte signaling and fate exist. This review will discuss different potential cell metabolism pathways involved in shaping T lymphocyte function and differentiation. We will also provide a general framework for understanding how tumor microenvironmental metabolism, associated with hypoxic stress, interferes with T-cell priming and expansion. How T-cell metabolism drives T-cell-mediated immunity and how the manipulation of metabolic programing for therapeutic purposes will be also discussed.

## Introduction

Accumulating evidence indicate that the resolution of antigenic aggression (i.e., cancer and viral infection) requires the coordinated response of various heterogeneous immune cell types to a range of physiological and pathological signals to regulate their proliferation, migration, differentiation, and effector functions (1). One of the mechanisms by which immune cells integrate these signals is through the modulation of their metabolic activity (2). The diverse functions of the immune system require several bioenergetic processes. The metabolic pathways of oxidative metabolism fuels effector functions of immune cells (3, 4). In this regard, effector T cells have been reported to metabolically reprogram and upregulate glucose, amino acid, and iron uptake to support the synthesis of the new macromolecules necessary for T-cell clonal expansion and effector function (5, 6). While metabolite fluctuation caused by host immune cells is at present an active area of study, the pathways involved in T-cell activation and differentiation, in class switching effector T cells into memory T cells and how metabolism regulates immune function and plasticity remains unclear and very challenging. It is well established that low oxygen (O_2_) availability is a hallmark of most solid tumors in which infiltrating leukocytes experience severe hypoxia once away from nurturing blood vessels (7, 8). Therefore, it is not surprising that pathways of hypoxic stress response, largely governed by hypoxia-inducible factors (HIF), are highly relevant to the proper function of immune cells (9). There is at present a tremendous increase in studying how immune cells function in terms of their intracellular metabolism and how these metabolic pathways affect the phenotype and activation of immune cells. Nevertheless, although how hypoxia regulates T-cell metabolism and survival is relatively known (10), very little is known about how hypoxia-associated metabolism influences T-cell activation and effector function. Clearly, the metabolic activity of T cells in the context of tumor microenvironment, its heterogeneity, and complexity is an important consideration in immunotherapy, as activated T cells go from an Oxygen and nutrient-rich environment in the blood vessels to a comparatively Oxygen and nutrient-poor environment of hypoxic tumors.

Furthermore, reprograming of immune cell metabolic configuration (11) could be profoundly influenced by the metabolic microenvironment (12, 13). Human deregulation of systemic metabolism, such as obesity development, is often associated with altered immune cell metabolism (14). In obesity, the expanded adipose tissue (AT) is accompanied by immune cell infiltration (15). In turn, the resulting low-grade inflammation status constitutes an important initiator of the microenvironment favorable for tumor development. In fact, AT is recognized now as potent endocrine organ by secreting pro-inflammatory cytokines [such as tumor necrosis factor-alpha (TNF-α) and interleukin-6 (IL-6)] and adipokines (such as leptin) in the tumor microenvironment, with great significant impact on both tumor and immune cells (16).

Obviously, a deeper understanding of these metabolic-related issues and their influence on T-cell function may offer new therapeutic options in future to boost the efficacy of treatments in particular of cancer disease.

## T-Cell Metabolism is a Key Determinant in T-Cell Fate, Differentiation, and Function

**Figure 1:**
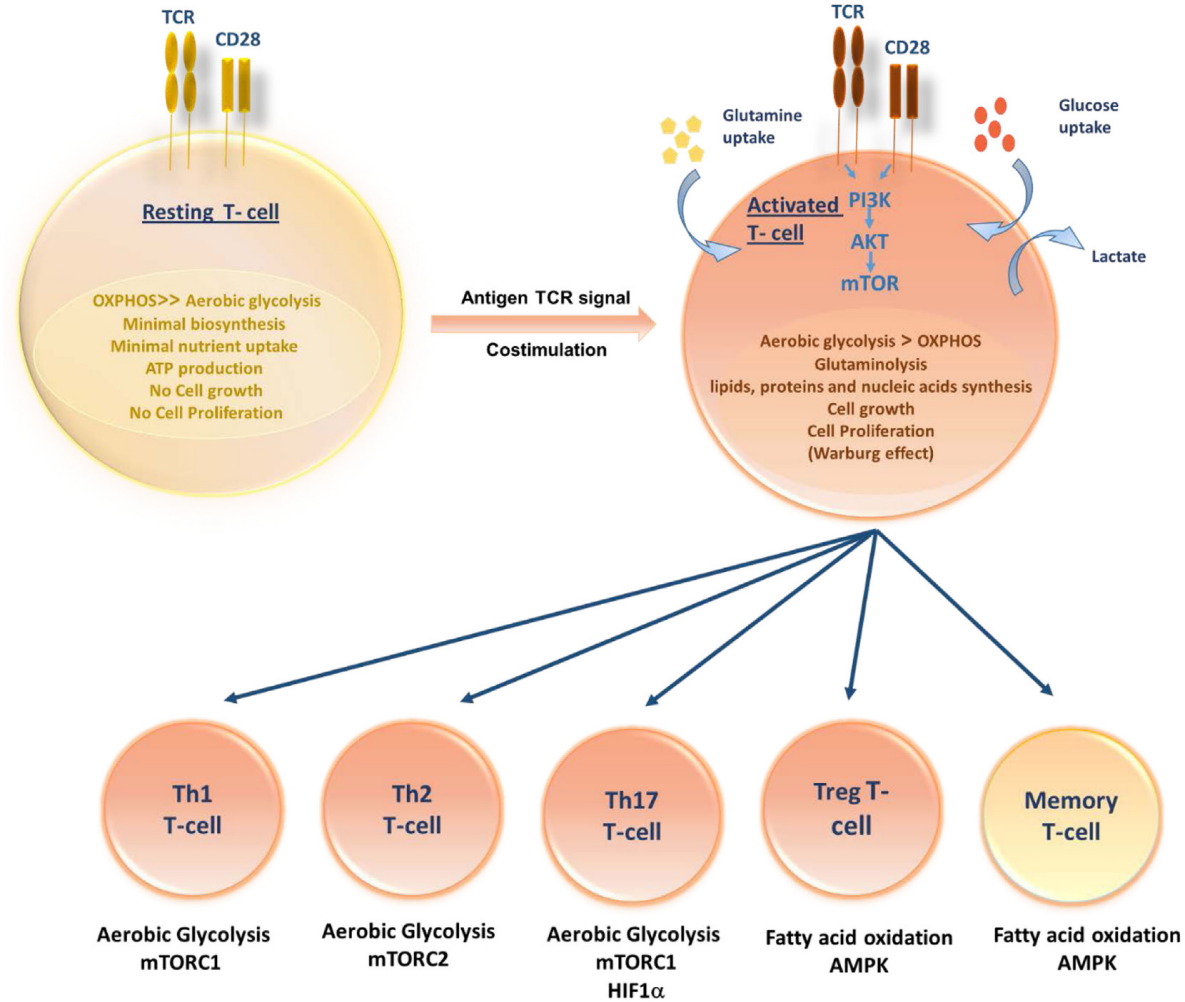
**Role of T-cell metabolism in shaping T-cell differentiation**. Upon activation, activated T cells rapidly switch to aerobic glycolysis, increase glucose and glutamine uptake and biomolecules synthesis, supporting cell growth and proliferation. Differentiation of activated T cells into different subsets is due to several metabolic and signaling pathways. While Th1, Th2, and Thl7 principally rely on aerobic glycolysis, Tregs and memory T cells upregulate fatty acid oxidation controlled by AMPK.

The pathways that control immune cell function and differentiation are intimately linked to cell metabolism. Like other cells, T cells use glucose as their primary fuel source for generation of adenosine triphosphate (ATP) and it is necessary for their survival, growth, activation, proliferation, and cytokine production (17). During quiescent state, resting T cells (naive, memory, and anergic T cells) have low metabolic requirements that serve to fuel basal energy generation and replacement biosynthesis. Resting T cells, therefore, have a metabolic balance that favors energy production over biosynthesis, and appear to oxidize glucose-derived pyruvate along with lipids and amino acids via the TCA cycle. Unstimulated thymocytes generate approximately 96% of their ATP via mitochondrial oxidative phosphorylation (OXPHOS) (18). Following activation, stimulated T cells must rapidly grow, divide, and exert effector function. Once T cells divide, they differentiate to different T-cell subsets, each switching on distinctive metabolic pathways (Figure 1). Proliferating cells must increase ATP production and acquire or synthesize raw materials, including lipids, proteins, and nucleic acids (19). To do so, proliferating T cells actively reprogram their intracellular metabolism from catabolic mitochondrial OXPHOS to glycolysis and other anabolic pathways. Despite the more rapid turnover of ATP via glycolysis when compared to OXPHOS, aerobic glycolysis is much less efficient in terms of the amount of ATP generated per molecule of glucose consumed. Furthermore, during this metabolic reprograming, glycolysis increases along with glutamine oxidation (20). Lipid oxidation, however, decreases sharply, and lipid synthesis rather than oxidation is favored. T-cell activation is thought to drive principally increased glucose uptake accompanied by a rapid increase in Glucose transporter-1 (Glut-1) expression with upregulating aerobic glycolysis, providing precursors for biomass synthesis (21). However, when rates of glucose and glycolysis are low, mitochondrial ATP production plays an important role in activated T-cell function (22, 23). If inadequate nutrients are prolonged, this may lead to T-cell anergy (24, 25) or T-cell death. In addition to glucose metabolism, T cells utilize glutaminolysis to meet their increased bioenergetic and biosynthetic demands (26). Although the mitochondrial pool of acetyl-CoA is mainly derived from the breakdown of glucose, glutaminolysis serves to replenish intermediates of the TCA cycle that are redirected into biosynthetic reactions, a process known as anaplerosis (27, 28). Furthermore, functionally distinct T-cell subsets require distinct energetic and biosynthetic pathways to support their specific functional needs (22). Recently, it has become increasingly apparent that T-cell activation does not lead to a uniform metabolic reprograming in all conditions. Regarding metabolic differences between activated T-cell subsets, CD4+ T cells have greater metabolic flexibility with respect to energy-rich substrates than effector CD8+ T cells. Activated CD4+ T cells are thought to increase both glycolysis and OXPHOS; however, CD8+ T cells increase glycolysis but may not increase OXPHOS. Thus, CD8+ T cells are exquisitely sensitive to the availability of glucose. A lowering in glucose concentrations prevents CD8+ T-cell activation, proliferation, and interferon-γ (IFN-γ) production (29).

An effective T-cell activation requires two signals: an antigen-specific signal induced by the T-cell receptor (TCR) and a co-stimulatory signal delivered by the surface receptor CD28. Activation of TCR along with CD28 co-stimulation triggers the induction of phosphoinositide-3-kinase (PI3K)-dependent activation of Akt pathway (30). It has been reported that the induction of glucose metabolism during T-cell stimulation is dependent on the activation of PI3K. In addition, Akt has been shown to control changes in glucose transport and metabolism (31). Therefore, it is conceivable that Akt is the PI3K-dependent factor controlling glucose metabolism downstream of co-stimulatory signals. The Akt pathway activation upregulates Glut-1 expression and stimulates its localization to the plasma membrane of T cells, so facilitating increased glucose uptake (21). Furthermore, Akt controls the activation state of the mammalian target of rapamycin (mTOR) (32). In turn, mTOR modulates rates of protein synthesis that support cell growth and effector functions (33). Besides, mTOR has recently been shown to play a crucial role in the commitment to distinct differentiation pathways by CD4+ and CD8+ T lymphocytes. It orchestrates the induction of anergy, the differentiation fate of CD4+ T cells into inflammatory and regulatory subsets, the development of CD8+ memory T cells and the regulation of T-cell migration patterns (24, 34). In activated T lymphocytes, the metabolic transition toward increased glycolysis is not only associated with mTOR induction but also associated with the expression of additional transcription factors, including Mycand HIF-1α able to enforce metabolic phenotypes in effector T cells appropriate for their function (35, 36). The role of hypoxia via HIFs in the regulation of T-cell metabolism and differentiation has been poorly studied in different T-cell subsets. A few studies have shown that Hypoxia via HIF-1α plays a vital role in the differentiation of Th17 and T regulatory cells. Among different T-cell subsets, Th17 are highly glycolytic and rely heavily on glycolysis. The upregulation of HIF-1α resulted in high levels of glycolysis in Th17 cells by increasing the expression of Glut-1 and PKM (37). HIF-1α was shown to shift from Th2 to Th17 cells differentiation by activating transcription of RORγt and directly upregulating IL-17 gene (38). Simultaneously, HIF-1α inhibits the T regulatory cells differentiation by targeting Foxp3 (39) for proteasomal degradation and, hence, shifting from T reg to Th17 cells (38).

## Tumor Microenvironment and Its Role in Modulating Immune Cell Metabolism

### Altered T-Cell Metabolism in Cancer

The ability of T cells to transition from a naïve to effector or to a memory phenotype is profoundly determined by metabolism and the metabolic program varies to match the T cells subset in order to enable cell survival and function. However, tumor microenvironment profoundly influences T-cell metabolism, and in consequence the host immune response. Increasing pieces of evidence suggest that T cells in the context of established progressing cancers exhibit anergic or exhausted state leading to T cell-intrinsic dysfunction. This blunt of T-cell function induced-tumor escape from immunosurveillance is ascribed to changes in the tumor cells themselves and to immunosuppressive factors in the tumor microenvironment. Regarding their metabolic state and effector functions, T cells can exert both tumor-suppressive and tumor-promoting effects (40–43). Thus, manipulating tumor cells and tumor microenvironment may provide a therapeutic approach to improve T-cell metabolism and to enhance their functions. Currently, how hostile tumor microenvironment can affect T-cell immune responses by altering the resulting cellular metabolism and leading to immunosuppression is attracting major attention in particular the ability of tumors to subvert normal immune regulation to their advantage. Tumor microenvironment in patients with a variety of solid tumors has revealed that a major subset of tumors shows evidence of increased immunosuppressive cells, such as regulatory T cells (Treg), immunosuppressive cytokines derived as from Treg and from tumor cells and poorly functional effector T cells expressing molecules capable of preventing T-cell activation (44, 45). It is important to underline that CD28 family, such as CTL-associated antigen-4 (CTLA-4), and programed death-1 (PD-1), is highly expressed on exhausted T cells. Engagement of B7 family members with CTLA-4 and PD-1 leads to inhibition instead of activation of T cells. Expression of these inhibitory receptors may restrain T cells from correctly remodeling their metabolism and, hence, dampen their function (46). Accumulating data suggest that the immunosuppressive metabolic environment could be further enhanced by tumor expression of inhibitory ligands for PD-1 and CTLA-4 that inhibits the upregulation of glucose and glutamine metabolism following TCR engagement and co-stimulation (47). Such inhibitory effect leads to T-cell inactivation and escape of tumor cells from immune attack (48, 49). Targeting exhausted T cells with the aim of enhancing glycolysis may be a way to reactivate these cells. Recent preliminary observations indicate that checkpoint blockade therapy alters the metabolic balance between tumors and their infiltrating T cells (48).

### Metabolic Reprograming of Tumor Cell and T-Cell Nutrient Deprivation, the Warburg Effect

Cancer cells require increased rates of glucose and glutamine consumption allowing them to produce energy (ATP) and the nucleotides, amino acids, and lipids required for proliferation. This phenomenon of metabolic reprograming, recognized as “Warburg effect,” is now considered one of hallmarks of cancer cells (50–52). Cancer is also a disease of altered metabolism. Warburg’s aerobic glycolysis is not only a feature of cancer cells. The same reprograming to aerobic glycolysis is exhibited, for instance, by highly proliferating normal cells, such as activated lymphocytes (19). Recently, it has been hypothesized that immunosuppression in the tumor microenvironment is at least in part driven by the inability of T cells to acquire the nutrients to support their metabolism (53). In this regard, several lines of evidence suggest that the altered T-cell function leading to immune escape is due in part to the ability of tumors to subvert normal immune regulation to their advantage (54). It is conceivable that the exacerbated glycolytic phenotype of tumors may contribute to a strongly immunosuppressive microenvironment. In this regard, tumor cells with increased consumption of glucose and glutamine may cause nutrient deprivation to effector T cells and the subsequent acquisition of anergic phenotype, leading to immunosuppression. It should be noted that oncogenic mutation BRAF V600E (55) induces constitutive activation of the MEK–MAPK pathway, leading to enhanced tumor cell proliferation, suppression of OXPHOS, and a highly glycolytic rate (56, 57). Because glucose and glutamine are crucial for T-cell differentiation and function, and their depletion impairs T cells cytolytic activity as well as interferon-γ (IFN-γ) production (58), it is most likely that the highly glycolytic phenotype of BRAF V600E melanoma may be associated with an immunosuppressive microenvironment.

### Effect of Hypoxic Tumor Microenvironment on T-Cell Metabolism and Function

Hypoxia-inducible factors are master regulators of tumor and T-cell metabolic responses to hypoxia (7). Hypoxia was shown to inhibit human T-cell proliferation, cytokine production, and function due to increased lactic acid production by tumor cells within the tumor microenvironment (59). Hypoxic microenvironment has diverse effects on T-cell responses and several oxygen-dependent and oxygen-independent stabilizers of HIFs, mostly HIF-1α has been identified in T cells (8). TCR-mediated stabilization of HIF-1 has been reported following antibody-mediated engagement of TCR/CD3 via the PI3K/mTOR pathway leading to increased HIF-1α protein synthesis (60). TCR-activated T cells also increased HIF-1α mRNA synthesis by mechanisms involving protein kinase C (PKC) and Ca (2+)/calcineurin (61). Independently of TCR stimulation, HIF-1α mRNA is augmented in T cells in the presence of TGF-β and/or IL-6 by a mechanism involving STAT3 (38). Hypoxia can interfere with the differentiation and function of immune cells by modulating the expression of co-stimulatory receptors and the type of cytokines produced by these cells. It has been reported that HIF-1 is involved in the upregulation of several co-inhibitory and co-stimulatory receptors, such as PD-1, CTLA-4, LAG3, CD137, and OX40, on the surface of hypoxic or VHL-deficient T lymphocytes (62). Interestingly, the discovery that PD-1 and CTLA-4 are direct transcriptional targets of HIF can be of utmost importance since they represent key pathways of resistance to immunity. Moreover, under hypoxic conditions, TCR activation results in an increase in FOXP3 on CD4 T cells in a TGF-β-dependent manner, thereby impacting the differentiation toward Tregs (39). The role of HIF-1a has also been identified in promoting the recruitment of Treg cells to the tumor microenvironment via over-expression of cytokines and chemokines, such as TGF-β CCL28 by hypoxic tumor cells (63). The effects of hypoxic stress on the killing functions of CD8^+^ T cells have been analyzed by several groups. Recently, deletion of *Vhl* in CD8^+^ T cells, which resulted in constitutive expression of HIF-1 and HIF-2, delayed CD8^+^ T-cell differentiation into effector cells but increased their cytotoxic functions and more interestingly several immune checkpoint receptors were increased in both HIF-1- and HIF-2-dependent manner (62). Hypoxia has been shown to both increase and inhibit T-cell responses. Roman et al. showed that hypoxia increased the secretion of cytokines, such as IFN-α by CD4 effector cells (64). By contrast, hypoxia decreased IL-2 from T lymphocytes resulting in an impaired immune response (65). Similarly, hypoxia was shown to decrease activated T-cell numbers by increased apoptosis (66). On the other hand, hypoxia was demonstrated to increase the survival of antigen-specific T cells through upregulation of adrenomedullin (67). Further studies on how hypoxia-mediated glycolysis may regulate especially T-cell differentiation and function in a tumoral context are much needed. Moreover, we still need to dissect the potential roles of both HIF-1α and HIF-2α in the regulation of T-cell metabolism and function.

### Stromal Endothelial Cell Role in Modulating Immune Cell Metabolism and Function

Endothelial cell metabolic activity has a significant effect on immune cells action and recruitment inside the tumor site (68). PHD inhibitors as fumarate/succinate produced by tumor cells during tricarboxylic acids cycle, is a potent way of HIF-1α stabilization (69). Moreover, several metabolites produced influence macrophages polarization from M1 to M2 phenotype (70). Indeed, the increased arginase 1 levels in macrophages depend upon the lactate acidification of the tumor microenvironment, thus, reducing the efficacy of the immune response (71). Moreover, lactate is efficiently pro-inflammatory through IL-17A secretion and inhibition of CTLs (72). Inflammation also consumes glucose, influencing Treg cell differentiation in inflammation (71). A consequence of the metabolic signals is the expression in the tumor microenvironment of immune checkpoint ligands PD-L1 and PD-L2 (73, 74).

Besides the direct tumor cell expression of PD-1 ligands, which is one of the most potent immune checkpoints to counteract in order to permit CTL and NK cells activity, the endothelial metabolism might rule first their recruitment and participate to their inactivation (75). Stromal and immune cells adopt their metabolism to optimally exert their distinctive role as support cells in particular conditions, in which each of these stromal and immune cells must fulfill specialized functions. Overall, these findings highlight a potential consideration for future immunotherapy.

## Cell Metabolism in Tumor and Non-Tumor Cells

Normal resting cells produce ATP through an energetically efficient metabolic program that serves to meet the energetic requirements of maintaining homeostasis (76). During proliferation, normal cells activate metabolic pathways to generate sufficient energy to support cell replication, and to satisfy the anabolic demands of macromolecular biosynthesis of cell reproduction (77). The aerobic glycolysis shift of proliferating cells is perfectly controlled by signaling and transcriptional circuitry that modulates cell growth. Yet, this metabolic boost is primarily fueled by glucose and glutamine and correctly maintained by a variety of checkpoints (78).

During malignant transformation, cancer cells show atypical metabolic characteristics that support inappropriate cell proliferation. Rapidly proliferating cancer cells is marked by increase in glucose uptake and consumption (79), which is metabolized to lactate under aerobic glycolysis independently of oxygen level and mitochondria damage, referred to as “Warburg effect.” Glutamine is another nutrient and important source of nitrogen, highly consumed by cancer cells. The growth and persistence of tumor cells benefit of increased flux of glycolytic and glutamine intermediates, supporting macromolecules biosynthetic pathways (80). It should be noted that both genetic and non-genetic factors can also directly modulate the metabolism of cancer cells, although many of the metabolic alterations are largely similar to those in normal proliferating cells (81).

Several mutations that activate oncogenes or inactivate tumor-suppressors genes impart cancer cells with the ability to disrupt multiple metabolic signaling pathways. Mutation of PI3K has been usually associated with cancer metabolism (82). This alteration activates the PI3K/AKT/mTOR pathway that results in stimulating glucose uptake and glycolysis by affecting activities of key glycolytic enzymes, such as hexokinase (HK) and phosphofructokinase (PFK) (83, 84). Furthermore, mTOR indirectly causes stabilization of HIF-1 (85). HIF-1 activates PDK, which inactivates the mitochondrial pyruvate dehydrogenase complex and thereby inhibits the entry of pyruvate into the TCA (86). In addition, mutation of c-Myc oncogene also has been described to enhance transcriptional activities of key enzymes of glycolysis and glutaminolysis in cancer cells (87, 88). More importantly, the tumor-suppressor p53 has been reported to support OXPHOS via SCO2 and to suppress glycolysis by enhancing TIGAR (89).

## Obesity, Low-Grade Inflammation, and Cancer

### Immune Response in Obesity

Several lines of evidence revealed that obesity can cause impairment of immune functions and metabolic homeostasis inducing chronic inflammation of white adipose tissue (WAT) and the resultant increased circulating concentrations of inflammatory markers (90). There is accumulating evidence that expanded AT results in increased serum levels of cytokines, such as TNF-α and IL-6, in obese individuals (90). Subsequently, the elevated pro-inflammatory mediators induce the activation of IKKβ/NFκB and c-Jun NH(2)-terminal kinase (JNK) pathways, which are central coordinator of inflammatory responses (91). The inflammatory responses associated with obesity may have detrimental metabolic consequences (92). Furthermore, the chronic low-grade inflammation-associated obesity does not occur only in WAT but in many organs, including brown adipose tissue (BAT), pancreas, liver, brain, muscle, and intestine. However, WAT remains the most studied organ in terms of immune–metabolic interactions in obesity (93).

Adipose tissue is described as a heterogeneous tissue, which comprises multiple cell types, including a diverse array of immune cells. AT-resident immune cells exert a wide range of functions, but can roughly be divided into two groups: on the one hand, immune cells that drive AT inflammation and insulin resistance (IR) and, on the other hand, immune cells that protect against these pathologies (92, 94). The first group consists of pro-inflammatory cells [activated macrophages (AAMacs), neutrophils, Th1 CD4 T cells, CD8 T cells, B cells, DCs, and mast cells]; these cells produce TNF-α or IFN-γ, or induce the polarization of inflammatory M1 macrophages. Thereby, the AT-resident inflammatory immune cells drive T helper 1 (Th1) cell response. The second group comprises anti-inflammatory cells [M2 macrophages, regulatory CD4 T cells (Tregs), Th2 CD4 T cells, eosinophils, Group 2 innate lymphoid cells (ILC2s), invariant natural killer T (iNKT) cells], which produce IL-10, IL-4, or IL-13, and drive Th2 response (15, 95). In the steady state, crosstalk between adipocytes and immune cells contributes to the regulation of available balance of energy stores and energy expenditure for survival during times of starvation and pathogen challenge (96). There is extensive evidence showing that both macrophages and T cells are the most abundant leukocytes implicated in AT biology and appear to be at the center of obesity-related inflammation via a positive-Feedback Loop (97). Macrophages present significant plasticity and recent data showed that during obesity, resident adipose tissue-associated macrophages (ATAM) display a polarization state switch from an alternatively activated state macrophage (M2) to a more pro-inflammatory state macrophage (M1) (98). Yet, obesity is accompanied by a progressive infiltration into obese AT of activated (M1) macrophages (99). These M1 macrophages produce pro-inflammatory cytokines, such as IL-1b, IL-6, and TNFα, creating a pro-inflammatory environment that blocks adipocyte insulin action (100). It is likely that this phenotypic switch could be the key to propagating inflammation and IR.

Recently, the functional role of adaptive immune cells has been characterized in obesity (101). Cytotoxic T cells seem to regulate accumulation, differentiation, and activation of macrophages in obese AT. Although the exact mechanism is not well understood, it is suggested that effector CD8+ T cells recruitment in obese AT precede infiltration and accumulation of pro-inflammatory macrophages (102). CD8+ T cells produce IFN-γ that further promotes the recruitment and activation of M1 macrophages. In turn, M1 macrophages enhance antigen presentation to CD4+ T cells and induce Th1 cell polarization and proliferation (103, 104). T-bet-dependent CD4+ Th1 cells produce TNF-α and IFN-γ to further potentiate IR in WAT (103, 105). How WAT inflammation is triggered is not completely understood, but it is suggested that lipotoxicity (106), endoplasmic reticulum (ER) stress, and Toll-like receptor activation (107) are involved. Collectively, regarding the dynamic interactions between immune cells and adipocytes in obesity, the CD8+ T cell/classically activated macrophage pathway in AT appears to be critical for establishing the pro-inflammatory status. Therefore, intervening with ATM, T cells directly may represent a therapeutic target for improving obesity-induced IR.

### Obesity-Induced Inflammation and Cancer

Adipose tissue has been proposed to contribute to the low-grade inflammation and to mediate the responses linking obesity to cancer. Both adipocytes and infiltrating immune cells coordinate to provide tumorigenic and pro-invasive microenvironment conducive to metastatic progression (108). Moreover, it has been suggested that the inflammatory cells and cytokines found in tumors are more likely to contribute to immunosuppression than they are to mount an effective host anti-tumor response (109). In fact, AT is recognized now as potent endocrine organ by secreting pro-inflammatory cytokines (such as TNF-α and IL-6) and adipokines (such as leptin) in the tumor microenvironment (16). Adipokines secreted from cancer-associated adipocytes likely form a key component of the paracrine signaling in the tumor microenvironment, favorable for tumor growth (110–112). Additionally, leptin plays critical roles in the regulation of glucose homeostasis and has been implied as an effector of obesity-induced changes in tumor and stroma cells (113). Leptin intracellular signals through its receptor OBRb involve the activation of several pathways commonly triggered by many inflammatory cytokines [JAK2/STAT; (MAPK)/extracellular regulated kinases 1 and 2 (ERK1/2) and PI3K/AKT1, and non-canonical signaling pathways: PKC, JNK, and p38 MAP kinase] (114). Within cancer development, chemotactic factors are produced causing an influx of inflammatory cells. Furthermore, these activated cells within the tumor secrete pro-inflammatory cytokines, IL-1, IL-6, IFN-γ, TNF-α, and IL-17 (115). In turn, stimulated tumor cells by these cytokines induce the production of CCLL22, a chemokine that then recruits Tregs into the tumor (16, 116). Hence, therapeutic modulation of obesity-associated pathways could be an interesting target to improve immune function in cancer.

## New Metabolic Targets and Recent Approaches to Modulate Tumor Microenvironment Metabolism for Therapeutic Purposes

Conceiving new therapeutic approaches to target cancer metabolism is a real challenge and may prove difficult given that cancer cells share similar metabolic requirements than normal proliferating cells. Moreover, under metabolic rewiring, cancer cells generate thereby an important diversity and heterogeneity in the metabolic adaptation. Indeed, such heterogeneity is largely potentiated by both genetic and non-genetic factors (81).

Beyond glycolysis, targeting glycolytic enzymes has been exploited through therapeutic intervention that might specifically inhibit key metabolic steps associated with tumor growth, proliferation, and invasion. In fact, attenuation of rate-limiting steps of glycolysis is an attractive field of investigation for cancer metabolism therapy. Currently, several studies have been explored small molecules that block glycolysis by inhibition of key enzymes (117). In this context, an example of therapeutic opportunity is a glucose analog 2-deoxy-d-glucose (2-DG), which is a competitive inhibitor of glycolysis (118). Another glycolytic inhibitor is DCA that targets PDK1 and thereby reduce lactate production and enhance mitochondrial OXPHOS (119). Nonetheless, and given the recognition that metabolic reprograming potentiates nucleic acid synthesis to promote tumor proliferation, the pentose phosphate pathway (PPP) has been also targeted. 6-Aminonicotinamide (6-AN) is a molecule showing an inhibitory effect on 6-phosphate dehydrogenase (G6PD), a key enzyme of glycolytic shunt into PPP (120, 121). All these drugs showed promising results in pre-clinical studies and warrant further investigation for clinical trials confirmation.

Another metabolic target that has been explored is mTOR pathway, the activation of which is widely described to be involved in metabolic reprograming of many human cancers (122–124).

Intensive efforts are underway to directly target inhibition of HIF-1 activity for cancer therapy (125). Several directions were aimed to test this inhibiting efficacy on HIF-1 transcriptional activity and DNA binding, HIF-1 protein translation, and HIF-1 protein degradation. Accordingly, an increasing number of chemical inhibitors have been developed, respectively, to these mechanisms of action.

## Discussion

Accumulating evidence highlights how metabolic adaptations of stromal and immune cells determine their differentiation and function in health and disease. More importantly metabolic changes in these cells may contribute to the pathogenesis of cancer and inflammatory diseases. There is growing evidence that extracellular signals are required to upregulate lymphocyte nutrient uptake and metabolism to support cell growth, proliferation, and cytokine secretion. In this regard, the control of T-cell metabolism seems to be important in the effector/memory transition of T cells. This opens the possibility that the signaling pathways involved in lymphocyte metabolic control may be novel therapeutic targets. Despite the significant progress during the last decades in anti-tumor immunotherapy approaches, there is still a need for more innovative approaches integrating the simultaneous activation of immune effector cells in the context of metabolic tumor microenvironment. It is now clear that the behavior of lymphocytes and other leukocytes are controlled on many levels by internal metabolic properties. The elucidation of how the metabolic phenotype of activated T cells is influenced by the microenvironment is in this regard very challenging. It is also crucial to understand the implication of metabolism in reprograming these cells as well as the interplay between their metabolic and cellular signaling systems to improve the outcome of cancer immunotherapy. In addition, several examples illustrate the therapeutic potential of targeting metabolism in stromal and immune cells. Analyzing the metabolism of immune cell types is at present a challenging issue and may, therefore, have translational consequences. It is tempting to speculate that an in-depth study of the signaling pathways involved in lymphocyte metabolic control may lead to the discovery of several novel immunotherapeutic targets.

## Author Contributions

All authors listed, have made substantial, direct and intellectual contribution to the work, and approved it for publication.

## Conflict of Interest Statement

The authors declare that the research was conducted in the absence of any commercial or financial relationships that could be construed as a potential conflict of interest.

## Abbreviations

AAMacs, activated macrophages; ATM, adipose tissue-associated macrophages; ATP, adenosine triphosphate; BAT, brown adipose tissue; CTLA-4, cytotoxic T lymphocyte antigen-4; HIF, hypoxia-inducible factor; IFN-γ; interferon-γ; ILC2s, Group 2 innate lymphoid cells; iNKT, invariant natural killer T; IR, insulin resistance; mTOR: mammalian target of rapamycin; OXPHOS, oxidative phosphorylation; PD-1, programed death-1; PD-L1, programed death ligand-1; TCR, T-cell receptor; TGF-β, transforming growth factor beta; TME, tumor microenvironment; WAT, white adipose tissue.
